# A soft cable loop based gripper for robotic automation of chemistry

**DOI:** 10.1038/s41598-024-59372-1

**Published:** 2024-04-17

**Authors:** Lupo Manes, Sebastiano Fichera, Hatem Fakhruldeen, Andrew I. Cooper, Paolo Paoletti

**Affiliations:** 1 Leverhulme Research Centre for Functional Materials Discovery, Material Innovation Factory, University of Liverpool, Liverpool, L69 7ZD UK; 2 School of Engineering, University of Liverpool, Liverpool, L69 3GH UK

**Keywords:** Mechanical engineering, Chemistry

## Abstract

Robotic automation is proving itself indispensable in the modern Chemistry laboratory, but adoption is slowed down by the technical challenges of implementing such systems. This paper reports on a novel adaptive gripper mechanism that can easily and reliably grasp cylindrical and prismatic objects of various sizes with limited clearance required. The proposed design exploits the inherent compliance of a cable that is driven to fully envelope the target object. The cable is run through a rigid finger, allowing the loop to be placed around objects with minimal clearance required and to provide support for the object once the grip is complete. Thanks to the compliant nature of the mechanism, the gripper requires minimal control effort to complete a gasping task. A prototype of the gripper has been designed and built for chemistry automation tasks, where it showed very high grasp reliability with $\le 1\%$ grasp failures.

## Introduction

As scientific exploration progresses and the boundaries of knowledge expand, the search for new discoveries becomes increasingly complex^1^. This rings true for the field of Chemistry as well, where, in order to find new chemical compounds and materials meticulous research and experimentation is required. Because of this, the research community is putting significant effort in the development of mathematical and artificial intelligence models that can explore the chemical space and plan syntheses way more efficiently than any human researcher ever could^2,3^. Furthermore there has also been a stronger push for automation and robotics in Chemistry laboratories to increase the reliability and throughput of experimentation and produce more data to improve the AI outputs^4^, effectively creating a robotic scientist that can produce hypotheses, test them experimentally, and make adjustments based on the results.

Automation has been a part of the Chemistry lab for well over a century^5^, with devices being used to automate the more tedious tasks. As technology is improving alongside the workload on the researcher we are seeing more complex automated experimental setups that require less and less human input for the duration of the experiment. However, in spite of clear advantages, adoption of automation is slow due to significant barriers to entry. The main obstacle is often the amount of resources (i.e. time, money and space) required to set up an automated experiment^6,7^. These factors can be partially solved by adopting robotic manipulators, which provide a flexible and accurate motion platform capable of performing multiple tasks around the laboratory. The capabilities of the manipulator can be augmented even further by placing the robot on a mobile base. Indeed, recent reports showed that manipulators can be successfully used to automate both individual tasks and complete experiments. For example, King et al.^8^ developed an autonomous closed cell robotic system that can test genomics hypotheses generated by an algorithm. Fleischer et al.^9^ developed a closed robotic cell that can prepare liquid samples by using dual arm robot platform that can interface with electronic pipettes and measuring equipment. Jiang et al.^10^ developed a biomimetic robotic system based on an ABB YUMI dual arm cobot to carry out solid dispensing and metering tasks. Coley et al.^11^ developed closed reconfigurable robotic cell, utilising a Universal Robots UR5, that can synthesize organic compounds based on AI planning. Lim et al.^12^ developed a robotic cell for organic chemistry synthesis mimicking the human approach that can achieve the results of a junior chemist. Burger et al.^13^ developed a robotic system based on a Kuka IIWA robotic manipulator and mobile base that can operate an entire lab to complete experiments. These are but a few examples of the efforts to automate Chemistry experiments and display the trend to move from an industrial approach (closed cell) to cobots that can operate alongside humans. A robotic manipulator can be used as a versatile “gantry” to transport the samples from one step of the workflow to the next, but it can also carry out more complex tasks if required. For these reasons, robotic tools are becoming more and more commonplace in various areas of scientific research thanks to the increased throughput and repeatability of experiments^14^ they allow to achieve.

While robotic manipulators have significantly lowered the barriers to automation in Chemistry research, they are not without their challenges, particularly concerning the manipulation of laboratory supplies and equipment. Indeed, despite their adaptability, these robotic systems often face pain points associated with the intricacies of handling consumables in chemistry laboratory setting. This can be seen in the previous examples, where even though the manipulation problem is not stated clearly, it can be observed that a vast amount of the consumables have been customised for the application or used inefficiently to allow the robot to successfully complete the grasping task. This can slow down development and reconfiguration of the system as the laboratory and the supply need to be modified to better accommodate the robot’s capabilities.

The main deciding factor in determining what tasks the robotic manipulator will be able to attend is the design of its end-effector. As robots adoption increases across multiple fields new designs of grippers are needed to enable effective and efficient automation^15,16^. While a parallel jaw gripper can be made reliable and to complete a single tasks the design needs to be improved for tasks that require resilience to uncertainty. This is particularly apparent in agricultural^17^ and logistics^18^ automation, where the robot is required to manipulate objects that vary in size shape and orientation. In this effort to make robotic gripper more versatile we see larger focus on compliant mechanism and soft robotics^15,19^. Among these, Universal grippers stand out as they can easily manipulate object with very different geometries and physical properties with minimal sensing and control effort. The granular jamming gripper^20^, meshed pin array gripper^21^, and bio-inspired swallowing gripper^22^ are some examples of universal grippers designs. On the other end of the spectrum a lot of progress has been made on the control of anthropomorphic robotic hands like the shadow hand^23^ which has been used in conjunction with advanced sensing and control to achieve outstanding dexterity^24^.

Robotic manipulation in automated Chemistry has not yet received extensive attention from the robotic manipulation research community. For the most part it still follows the principles of industrial automation: a fully structured environment is created and parallel jaw grippers with bespoke fingers are used to manipulate the supplies, usually placed in bespoke fixtures. An example of this approach, widespread across Chemistry automation attempts, is fully documented in^25^ (chapter 3). In this work, the geometry of the fingers is optimised to create good mating surfaces with all the supplies required for the workflow, and the trays have been redesigned to better interface with the gripper. While this provides close to 100% reliability in manipulation, it makes the entire process costly to set up and difficult to reconfigure, as changing a vessel type might force a change of the design of the fingers and fixtures. This poses a hard limit to a research environment that will iterate over an experimental procedure multiple times before results are achieved. The finger shown in^25^ is but one example to demonstrate the general approach used in chemistry automation. In an environment such as a university’s chemistry lab where research is carried out, a “universal” gripper specialised for manipulation of the most common supplies would be ideal to enable automation, as it would increase the flexibility of a robotic system while requiring minimal onboarding effort. This particularly beneficial in these environments are eager to implement cobotic solution where the robot can augment the researchers’ capabilities without removing them completely from the laboratory^26,27^

**Figure 1:**
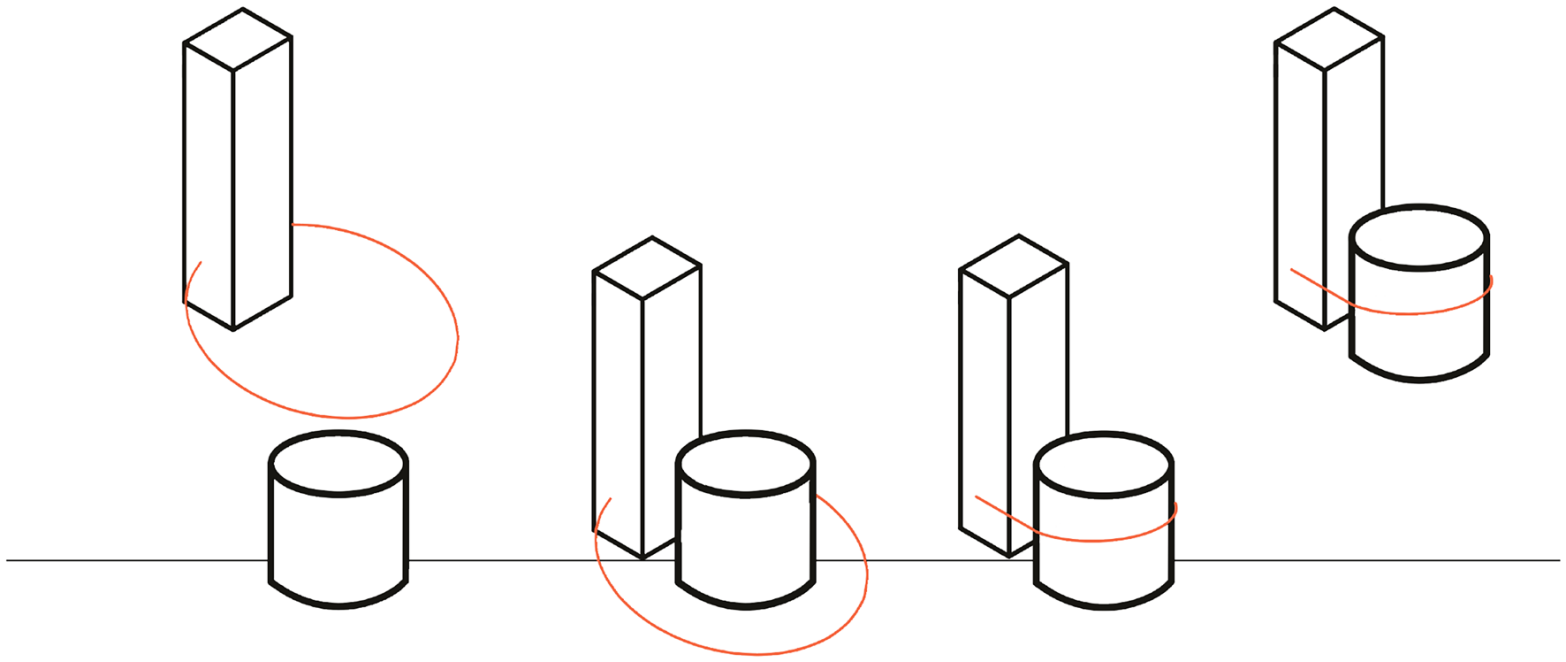
Schematics of the basic action sequence to complete a grasping task: (1) cable loop diameter is set to be larger than the diameter of the object to be grasped; (2) CLG is lowered; (3) cable loop is tightened around the object; (4) CLG is lifted.

In this paper, a novel adaptive grasping mechanisms that can heavily simplify the manipulation of laboratory glassware vessels in pick and place operation with minimal disruption to how the laboratory is presented.The Cable Loop Gripper (CLG) proposed in this paper employs a self-supporting loop of cable whose length, and hence diameter, can be altered by an actuator to seize any prismatic object. Cables are an extremelly common mean of actuation for robotic grippers design and have been used successfully in countless designs, from anthropomorphic hands^28,29^, to bio-inspired design^30,31^, since they can transmit power very efficiently, and requiring little space. The cable itself can be used as a grasping mechanisms, as shown by the Loop Gripper^32^ which uses many expanding loops of wire to manipulate comb materials. The mechanism we propose operates by being positioned around the targeted object and subsequently being pulled taut. In this manner, the cable will completely enclose the object of interest. The object is pulled towards a rigid finger to ensure that it is correctly positioned and remains stable during manipulation. A diagrammatic representation of a grasp is depicted in Fig. 1. This approach is extremely simple in execution but has not really been explored except in specialised medical applications such as the endoloop^33^.

**Figure 2:**
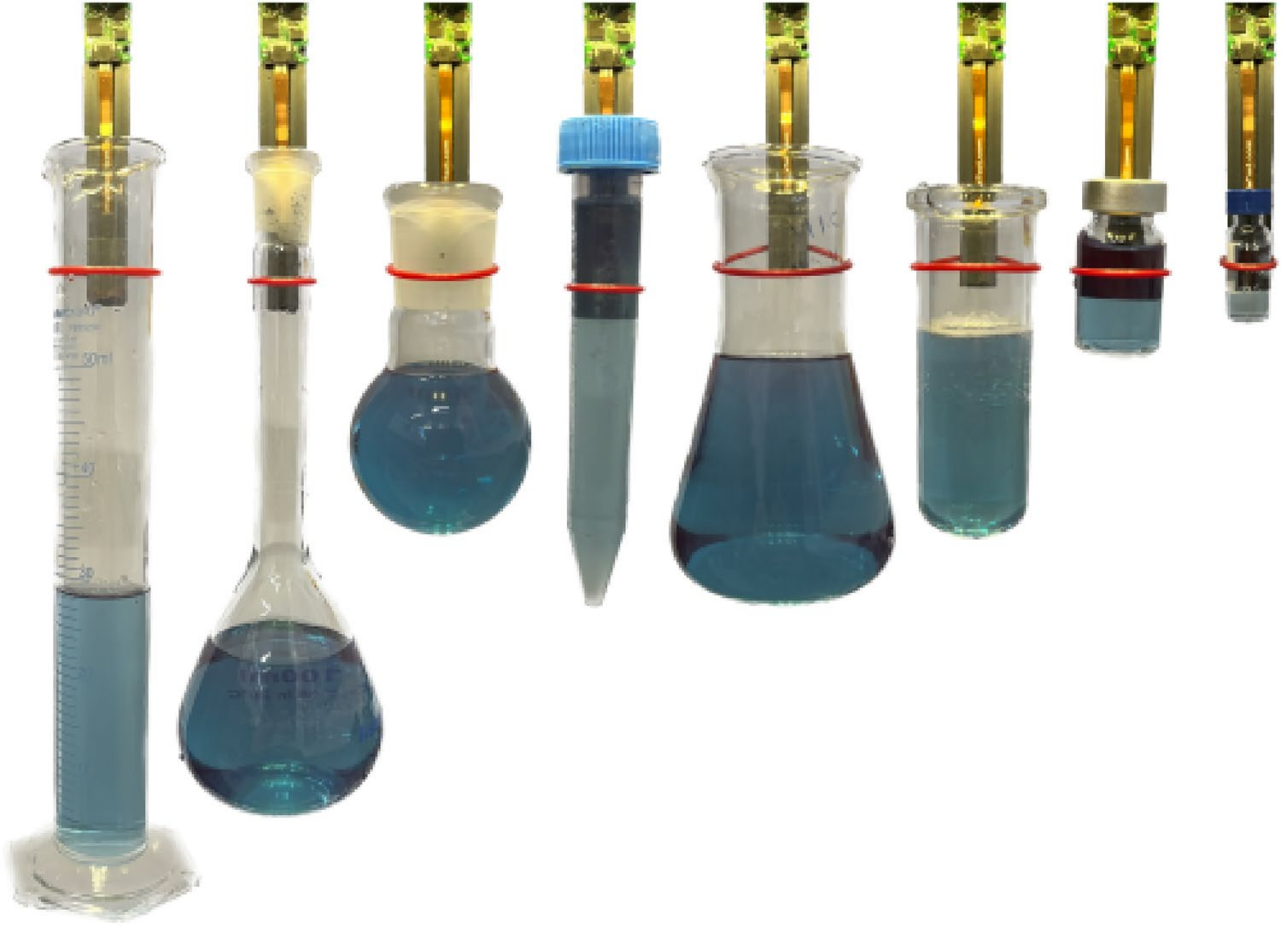
Cable loop gripper prototype grasping various Chemistry glassware.

. The mechanism exploits the inherent compliance of the cable to fully envelop object being manipulated, therefore leading to numerous advantages. The mechanisms can secure a diverse range of sizes, relative to its own dimensions, encompassing cylindrical and prismatic objects as shown in Fig. 2. Moreover, any grasp can be accomplished with minimal control effort, as long as the loop is positioned around the object and the cable is tightened to the requisite degree, ensuring a secure grip on the object. Furthermore, the gripper can effectively seize objects with minimal required clearance, as long as the cable can be manoeuvred around the object and the cable can attain the necessary tension. Finally, this grasping device can be easily miniaturised because the actuation components (motors, electronics, etc.) do not have to be placed at the grasping appendage, they can be moved away, and the cable routed the required position trough channels. This opens the possibilities to device with very small footprint, that can grasp object with little clearance required; enough space for the cable to be slid around the object and a small area for the finger to plunge into. It is also worth noting that the cable can approach the object from any angle and its not constrained to operating along an horizontal plane. Indeed the cable loop does not significantly deform under its own weight and therefore the gripper performance are only minimally affected by the overall orientation. All these properties make the device an ideal candidate as an end effector for a manipulator used for Chemistry automation, because the gripper can simplify the manipulation of test vials regardless of their size or how densely they are stored in trays. This removes at once all the needs for tailoring gripper design to specific workflows, and therefore can contribute to significantly accelerate robotic-assisted Chemistry.

## Design and methods

### Design considerations for a cable loop gripper

There are many factors that come into play during the design of a cable loop gripper despite its simple working principle. Here follow some insights regarding the main components of the system, their function and what factor need consideration during the design.

#### Cable

The cable is the key component of the proposed grasping mechanism, as it defines what the gripper is ultimately capable of handling. Therefore, the gripper performance is strictly linked to the physical properties of the cable. The design constraint that can be used to guide the selection of the cable are the minimum and maximum size of the object to be grasped, as well as the minimum clearance between objects in the given application. The clearance between objects set a hard limit for the diameter of the cable, as the cable needs to slot in between two adjacent objects. The maximum allowable cable diameter should be selected for the application, as the increased cross-section will favourably affect the stiffness of the material. The smallest object to be grasped will dictate the minimum bending radius required; too small bending radii may cause the cable to plastically deform, therefore rendering it unusable for further operation. Finally, the maximum object diameter will be dictated by the maximum size the cable loop can reach while withstanding its own weight, before bending too much to be useful. The amount of acceptable sag can be specified per application. The stiffness of the cable will affect the accuracy with which the mechanism can be controlled, and its compliance. Specifically, as the cable moves through a channel, it will be subject to different values of tension when pulled tight and compression when pushed wider. Softer materials will make accurate control of the loop size more challenging, as the cable will stretch and compress in the channel. On the other hand, softer materials are still preferable when grasping delicate objects; the increased compliance of the grasping mechanism still allows for solid and reliable grasp, without risking damaging the object. Finally, depending on the specific requirements of the application, other characteristics such as temperature or chemical resistance may need to be considered, especially when considering the original intended application for the device is Chemistry automation. Finally, one more feature to consider is the colour. This relevant when using vision feedback, as it greatly simplifies the image processing required to isolate the loop in the image.

While there many possible approaches to simulate the behaviour of a cable to select the optimal material for a given application^34^, they become overly complicated given the complexity of the material properties (especially for woven cables or cables made out of composites) and of frictional interaction between the cable, the finger and the grasped object. Due to this complexity, a more empirical approach is preferred and was used to create the prototype presented in this paper; testing a variety of cable materials and evaluate their performance directly in the mechanism during the initial prototyping phase.

#### Finger

The finger is the rigid component that allows the object to be manipulated securely by the robotic arm. Such component should be small enough to fit within the clearance between objects to be grasped. It also needs a feature to guide the cable into forming a self-sustained loop at the desired location, like channels embedded in the finger. Furthermore, the finger width should be as close as possible to the width of the most commonly grasped object to maximise the grip strength. Some feature should be embedded at the gripping end to constrain the grasped object movement when the cable is taught. Finally, the finger can embed sensors to provide real-time information during the grasping and manipulation phases.

#### Actuation

Actuation is needed to change the size of the cable loop. The actuation mechanism must be able to both pull and push the cable. While pulling the cable is trivial, the push action could be challenging depending on the stiffness of the cable. Linear actuators can be used to push and pull the end of an otherwise fully constrained cable or an active pulley and idler and pulley, in an arrangement similar to 3D printer extruder or TIG welder wire feed, pushing and pulling a cable into a channel that constraints its position in space but not the overall length.

#### Sensing

In order for the device to operate reliably, the device needs feedback over the size of the loop, the size of the object to be grasped and the grasping forces. A force sensor is placed behind a fingertip recess to provide force feedback during grasping. The output of the force sensor is used to control the motor current during grasping through a PI control loop.

A rotary encoder connected to the actuator can be used to estimate the current size of the cable loop by measuring how much cable has been pulled or pushed, neglecting potential slips between cable and extruder gears. However, vision is required the exact dimension and position of the cable loop relative to the finger compensating for slips, interactions between the cable and the channel, and loose tolerances required for the cable to move freely.

#### Control

Thanks to the simplicity of the proposed grasping mechanism, control hardware can be streamlined. The bare minimum requirement for the electronics is to be able to drive a servomotor, meaning supplying power to the motor, take encoder reading and run a PI loop. This approach has been chosen to control the grasping motion for the following reasons: i) the compliance of the mechanism makes it easy to maintain the desired contact force, ii) dynamic stability of the system during grasping is not an issue in the current setting, and iii) the PI controller can be easily run on an embedded device with minimal computational cost as well as being easier to tune regardless of the system model complexity. The output from the force sensor embedded in the finger is also used in conjunction with the output from the encoder during grasping to determine whether grasping was successful or not, by comparing the dimension of the cable during grasping against the expected one for the size of given object.

### Gripper prototype

**Figure 3:**
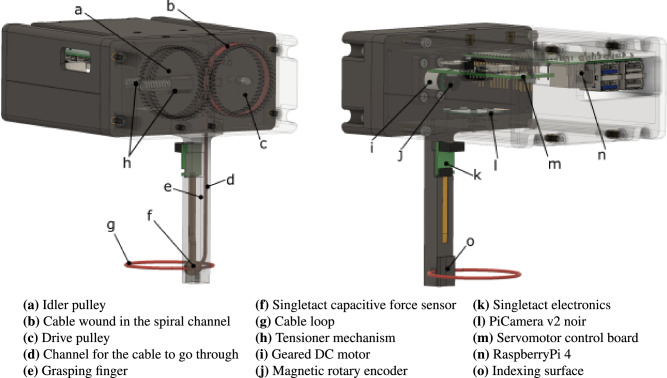
Front and back view of the cable loop gripper prototype with legend for all the key components.

A prototype device has been built to test the device in simulated Chemistry lab environment. A render of the prototype is shown in Fig. 3. In this device implementation, a 1.75 mm diameter TPU filament has been selected for the cable (g), as it provides solid wear and tear resistance indeed, the cable has not needed replacement over 6 months of intensive testing of the prototype device. and a small minimum bending radius (< 5 mm). The finger (e) is 15 mm wide to optimally trade-off between a small footprint for clearance and the capability to grasp object up to 50 mm diameter, therefore allowing it to grasp a wide variety of Chemistry glassware as shown in Fig. 2. The finger contains a channel for the cable (d) to be brought to the fingertip where the cable loop is formed. The finger has been 3D printed in PLA using an FDM machine (Ultimaker S5) to be able to manufacture the internal channels. The fingertip is not flat but has two surfaces (o) with a 160^∘^ angle to guide the grasped vial to the centre of the finger, and to prevent it from twisting during manipulation. A capacitive force sensor (f) has been placed underneath the fingertip to provide direct feedback on the grasping force. This specific design is capable of reliably lifting object between 10 and 60 mm diameter and up 100 grams in weight which makes perfectly suitable for manipulation of vials in batch chemistry workflows. The finger stems out of the control box, which contains the actuation, control and remaining sensing components. For actuation, the cable is wound around a drive pulley (C), and an idler pulley (a) is used to fully constrain the path of the cable and make sure it gets pushed through the channel. Idler and active pulleys move at the same speed to provide even contact force on both sides of the cable to drive it more reliably. A geared DC motor (i) drives the cable actuator and a magnetic rotary encoder (j) on the motor shaft provides feedback on the size of the loop. A camera ((l)) is placed in the box right above the cable loop to provide feedback on the size of the loop and the object to be grasped, as well as their relative position to one another. A servomotor control board (m) takes the reading from the encoder and force sensor and uses them to implement two separate PI control loops: one for controlling the size of the loop and one for controlling the gripping strength. Finally, A RaspberryPi single board computer (n) is used to process the camera feed and interface with the robotic manipulator.

**Figure 4:**
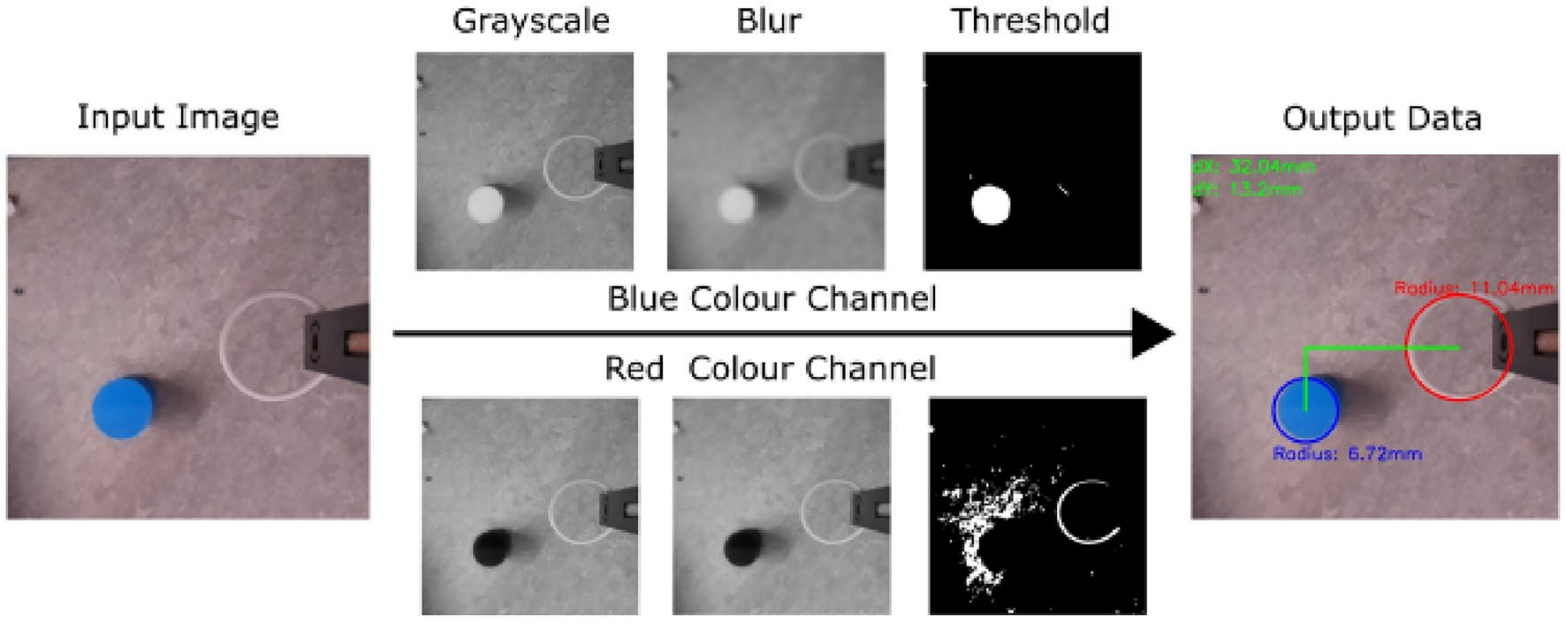
Visualization of the computer vision pipeline used to deect the cable and the manipulation target.

The vision module collects information about the cable and the target object using the eye in hand approach^35^. The module relies on an RGB-D camera mounted to the gripper body pointing at the cable loop. Because both the cable loop and the top-view of the Chemistry glassware are approximately circular, the processing of the camera outputcan be heavily simplified. The process for sizing the loop, shown in Fig. 4, consist of: isolating the red colour channel (as the used cable is red), apply a Gaussian blur to de-noise the image, apply a threshold filter and, finally, apply a Hough circle transform (^36^) to find the circle formed by the cable. An almost identical approach can be used to recognise the vials to be grasped. The vision sensing of the cable is used alongside the rotary encoder to account for visual occlusion during grasping and the higher polling ratio of the encoder. The depth sensing capabilities of the camera are needed to obtain the correct dimension of the vials. The vision feedback is also used for uncalibrated visual servoing that is robot agnostic, a principle that has been proved working^37^, but using its own tailor made visual pipeline. It provides a relative distance between the centre of the loop and the centre of the vial on the plane of the image, so that the loop can be placed vertically on top of the vial. the commands issued for servoing are relative to the position of the end effector.

The Robot Operating System (ROS) middleware framework has been used to develop a software package to control the gripper. This choice enables quick development and easy integration with most existing robotic systems through a standardised interface.

The prototype has been paired with a Panda (Franka Emika) 7 degree of freedom robotic arm to carry out functional testing of the mechanism, here follow the test setups.

### Reliability testing setup

**Figure 5:**
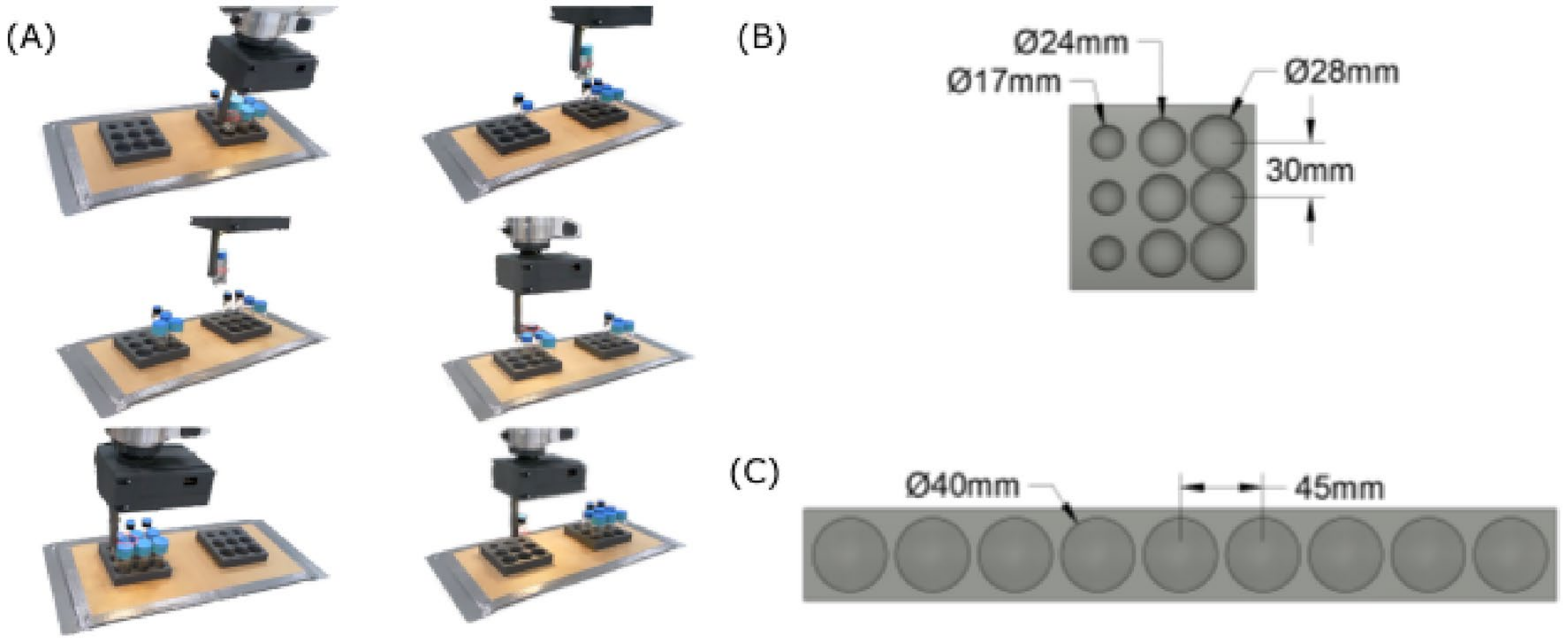
(**A**) snapshot from the reliability tests described in the reliability test setup section; (**B**) Tray storing the vials for the reliability and sorting test. (**C**) Tray used for the starting position of the vials during the sorting test described in the environment variability testing setup section.

This test is meant to simulate the most common action the robotic manipulator will carry out during a Chemistry experiment. A set of 9 vials of 3 different diameters, 3 of each, were moved between two different trays for a total of 1000 vials manipulations, the setup is shown in Fig. 5a. The trays, shown in Fig. 5b, kept the centre of the vials at the same distance of 30 mm from each another. This means a minimum clearance of 2 mm between edges for the 28 mm diameter vials. Blue stickers caps were used for the vials to aid the differentiation task of the machine vision algorithm. After moving the 9 vials from tray to tray, the arm was moved to the home position to simulate the running of an experiment. Every 2 full tray movements (18 manipulations), the cable loop radius was calibrated using the integrated camera to compensate for any play or slippage in the mechanism.

### Environment variability testing setup

The same vials and plate from the previous test were used for a sorting task. 9 vials of 3 different diameters were arranged in a line in random order inside oversized wells, where they could be placed in slightly different positions every run. The manipulator was then tasked with grasping each vial and place it into another plate where specific wells were used for each vial diameter (see Fig. 5c). Since the CLG was moved along the line of vials, it was capable successfully grasping all of the vials without failing on 5 repetitions of the test.

## Results and discussion

The prototype showed a failure rate of 0.8% and all the failures happens while grasping; no vial was lost while being moved around. The extensive reliability testing run for approximately 4.5 h, with each manipulation taking approximately 8 s from well to well, a speed comparable to what a human would achieve. Furthermore, the prototype has been able to seamlessly operate despite variation in the position of the manipulation target, up to ± 15 mm in the expected target position. While running the tests, it was also noted that the device provides good levels of compliance and inherent error recovery. The cable loop can deform and comply both during the pickup and deposition phase and, by doing so, recover from positional mistakes without dropping the grasped vial. Even in case of abrupt changes in the robot arm speed, the vials were not dislodged or dropped. In the case of a simulated power loss, friction in the system stopped the gripper from dropping its payload. It was also rather simple to calculate the position where the gripper can grasp from, since only one point of contact is required and the cable loop will easily deform to get to position as long as its diameter is larger than the target object diameter. One inconvenience of the design is that sometimes the cable can get caught on lips in the vessels, but this can be detected and solved by the robot-gripper system. The gripper was able to complete the test a reasonable speed with each pick operation taking less than 5 seconds including the visual seroving.

The gripper presented in this paper displays a mechanism that can reliably handle a wide variety of prismatic objects, passively compensating for small placement errors. It provides performance similar to a soft gripper, but it is easier to control and retains high payload and repeatability. The current design can be used effectively for the intended purpose of manipulating test vials of different sizes without having to modify any of the trays to account for the cumbersome parallel jaw grippers currently used for Chemistry automation. The finger provides a rigid point for the manipulation, while the cable can easily comply around the payload and provide secure grasping.

Although the device was designed for Chemistry automation, it could easily be adapted to other environments where small clearance grasping of prismatic objects is necessary. The main strength of the specific grasping mechanism proposed and implemented in this paper is its extreme versatility in manipulating chemistry glassware. On the other hand, cable loop-based grippers can not perform other tasks such as manipulation of large trays or objects with complex shapes such as retorts or condensers. This issue could be alleviated thanks to the small footprint the mechanism requires to be implement cable loop grasps, which makes it possible to integrate the proposed CLG into a finger of a parallel jaw gripper .

## Data Availability

All engineering design files and code are made available online on GitHub https://github.com/Liverpool-AlertLab/cable-loop-gripper.
